# Detectable 2019-nCoV viral RNA in blood is a strong indicator for the further clinical severity

**DOI:** 10.1080/22221751.2020.1732837

**Published:** 2020-02-26

**Authors:** Weilie Chen, Yun Lan, Xiaozhen Yuan, Xilong Deng, Yueping Li, Xiaoli Cai, Liya Li, Ruiying He, Yizhou Tan, Xizi Deng, Ming Gao, Guofang Tang, Lingzhai Zhao, Jinlin Wang, Qinghong Fan, Chunyan Wen, Yuwei Tong, Yangbo Tang, Fengyu Hu, Feng Li, Xiaoping Tang

**Affiliations:** Guangzhou Eighth People’s Hospital, Guangzhou Medical University, Guangzhou, People’s Republic of China

**Keywords:** 2019-ncov, laboratory test, blood viral RNA, pharyngeal swab, anal swab

## Abstract

The novel coronavirus (2019-nCoV) infection caused pneumonia. we retrospectively analyzed the virus presence in the pharyngeal swab, blood, and the anal swab detected by real-time PCR in the clinical lab. Unexpectedly, the 2109-nCoV RNA was readily detected in the blood (6 of 57 patients) and the anal swabs (11 of 28 patients). Importantly, all of the 6 patients with detectable viral RNA in the blood cohort progressed to severe symptom stage, indicating a strong correlation of serum viral RNA with the disease severity (*p*-value = 0.0001). Meanwhile, 8 of the 11 patients with annal swab virus-positive was in severe clinical stage. However, the concentration of viral RNA in the anal swab (Ct value = 24 + 39) was higher than in the blood (Ct value = 34 + 39) from patient 2, suggesting that the virus might replicate in the digestive tract. Altogether, our results confirmed the presence of virus RNA in extra-pulmonary sites.

The 2019 novel coronavirus (2019-nCoV), originally outbreaking from Wuhan China, has transmitted in an extremely short period to 25 countries and infected over 31 000 individuals as of Feb 06, 2020, causing an international alarm. Basic scientific research has achieved significantly in the investigation of viral origination [1,2], transmission and evolution [3], and unprecedented public health control actions in China have been activated and effectively prevented the otherwise dramatic spread. The 2019-nCoV virus seems more infectious in its public transmission capacity compared to the well-known 2003 SARS virus in spite of the unavailability of convincingly scientific evidence. The mechanism of viral transmission is still worthy of further exploration.

Currently, one urgent and critical challenge is to treat infected patients and save their lives. Several studies have roughly described the overall clinical features of 2019-nCoV patients [4,5]. However, the more specific and classified clinical characteristics of the infected patients still require further investigation, particularly for those with severe symptoms, which is roughly estimated to be approximately 15–20 percent of totally confirmed cases based on the local data in our hospital. Clinically, for those severe patients, the main symptoms of 2019-nCoV pneumonia are fever, decreased white blood cell and lymphocyte count, increased C reaction protein and abnormally expressed cytokines [6].

One remaining question to be resolved is whether the 2019-nCoV virus can replicate in extra-pulmonary sites, which might account for the deteriorated clinical manifestation. In this study, we investigated whether the patients with severe clinical symptoms exhibited special profiles of virus replication or/and distribution compared to those only with mild symptoms.

Patients, who were confirmed to be infected by the 2019-nCoV virus, were firstly enrolled in or transferred to Guangzhou Eighth People’s Hospital for treatment purposes. This study followed the guideline of the Ethics Committee of Guangzhou Eighth People’s Hospital. All blood, pharyngeal swab, and anal swab samples were collected for diagnostic purposes in the laboratory and our study added no extra burden to patients. Viral RNA was extracted with Nucleic Acid Isolation Kit (Da’an Gene Corporation, Cat: DA0630) on an automatic workstation Smart 32 (Da’an Gene Corporation) following the guidelines. Real-time reverse transcriptional polymerase chain reaction (RT–PCR) reagent (Da’an Gene cooperation, Cat DA0930) was employed for viral detection per the protocol. In brief, two PCR primer and probe sets, which target orf1ab (FAM reporter) and N (VIC reporter) genes separately, were added in the same reaction tube. Positive and negative controls were included for each batch of detection. Samples were considered to be viral positive when either or both set(s) gave a reliable signal(s).

All patients had pneumonia-based diseases but with diversified clinical manifestation. To simplify data analysis, the patients were only classified as either mild or severe clinical symptom groups based on the guideline newly released by Chinese government. Patients who were with at least one of the following symptom should be diagnosed to be severe case, 1) distress of respiratory with respiratory rate > = 30/min; 2) Oxygen saturation < = 93% in the rest state, and 3) arterial oxygen tension (PaO₂) over inspiratory oxygen fraction (FIO₂) of less than 300 mm Hg. In the blood detection cohort (Figure 1(A)), patients who had at less one serum sample measurement with the PCR method were included. In the 57, 6 cases were detected to be blood positive, all of them (100%) were severe in symptom requiring special care attention, and the blood of the rest 51 cases was without detectable virus in the blood, only 12 of them (23.5%) were severe cases. The ratio of severe symptoms between these two groups was significantly different (*p* value = 0.0001). In the anal swab cohort (Figure 1(B)), 11 of 28 cases were detected to be anal swab positive, 8 of them (72.7%) were with severe symptoms, which was significantly higher than that 4 (23.5%) of the rest 17 cases without detectable virus in anal were severe cases.

**Figure 1.**
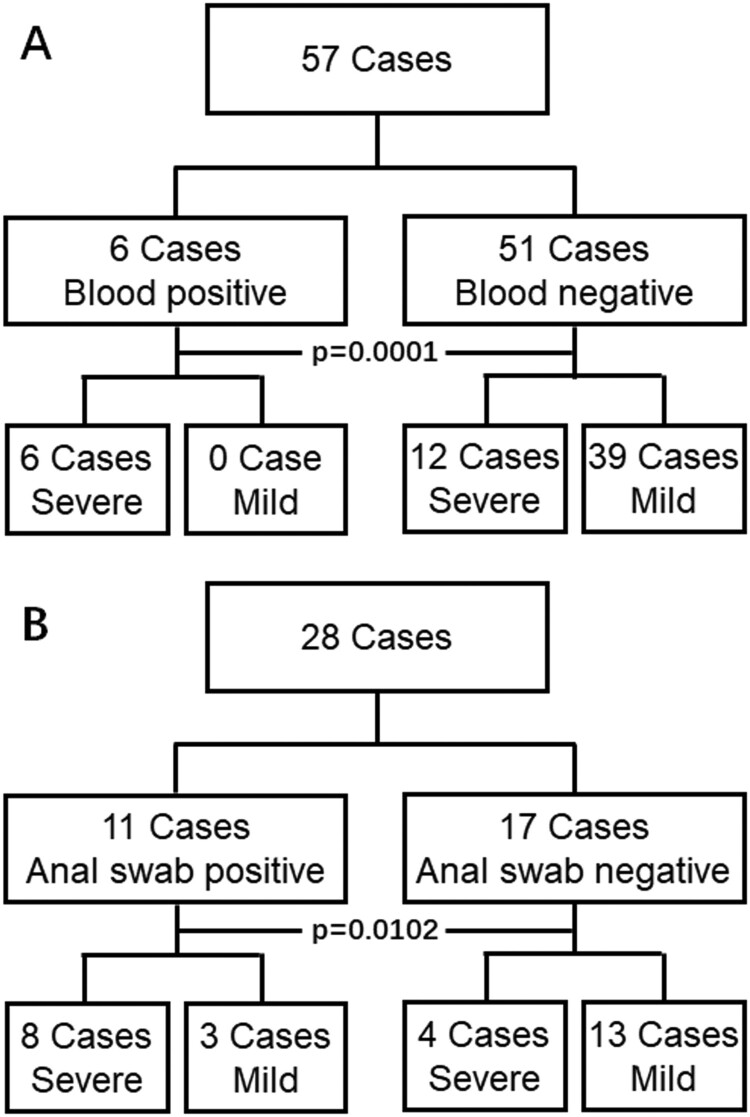
Results of 2019-nCoV viral RNA detection in the blood (A) and anal swab (B). Blood or anal swab positive represents viral RNA positive in at least one test of blood or anal swab samples from the same patient. Blood or anal swab negative represents viral RNA negative in all tests of blood or anal swab samples from the same patient. Severe represents that the patient is diagnosed as a severe symptom after expert consultation while mild represents the rest of the patients. *P* values are shown (chi-square test, two sides).

Fortunately, two cases with detectable virus both in blood and anal swab cohort were recorded. Patient 1 (Figure 2(A)) was admitted to ICU after enrollment evaluation and was highly suspected infection with 2019-nCoV because of his recent travelling from Wuhan and of confirmed pneumonia by radiographic diagnosis with 5-day fever and 1-day continuous dry coughing. He was then confirmed to be infected by the 2019-nCoV virus on illness day 6 by CDC. High concentrations of the viral RNA were detected in the pharyngeal swabs on illness days 5 (Ct = 17 + 25), 7, 8 (Ct = 25 + 26), and 11 (Ct = 15 + 25). In the blood, no viral RNA was detected on day 5 but the sample on day 6 gave a weak positive signal (Ct = Neg+39), and then the signal was gone again on day 8. On day 9, a low level of viral RNA (Ct = 36 + 41) was detected again in the blood. On day 12, the blood lost signal again. A high concentration of virus RNA (Ct = 23 + 27) was detected in the anal sample on day 13, on the day the 2019-nCoV virus was not detected in the pharyngeal swab. Unfortunately, he was transferred out to another hospital after an emergency expert consultation.

**Figure 2.**
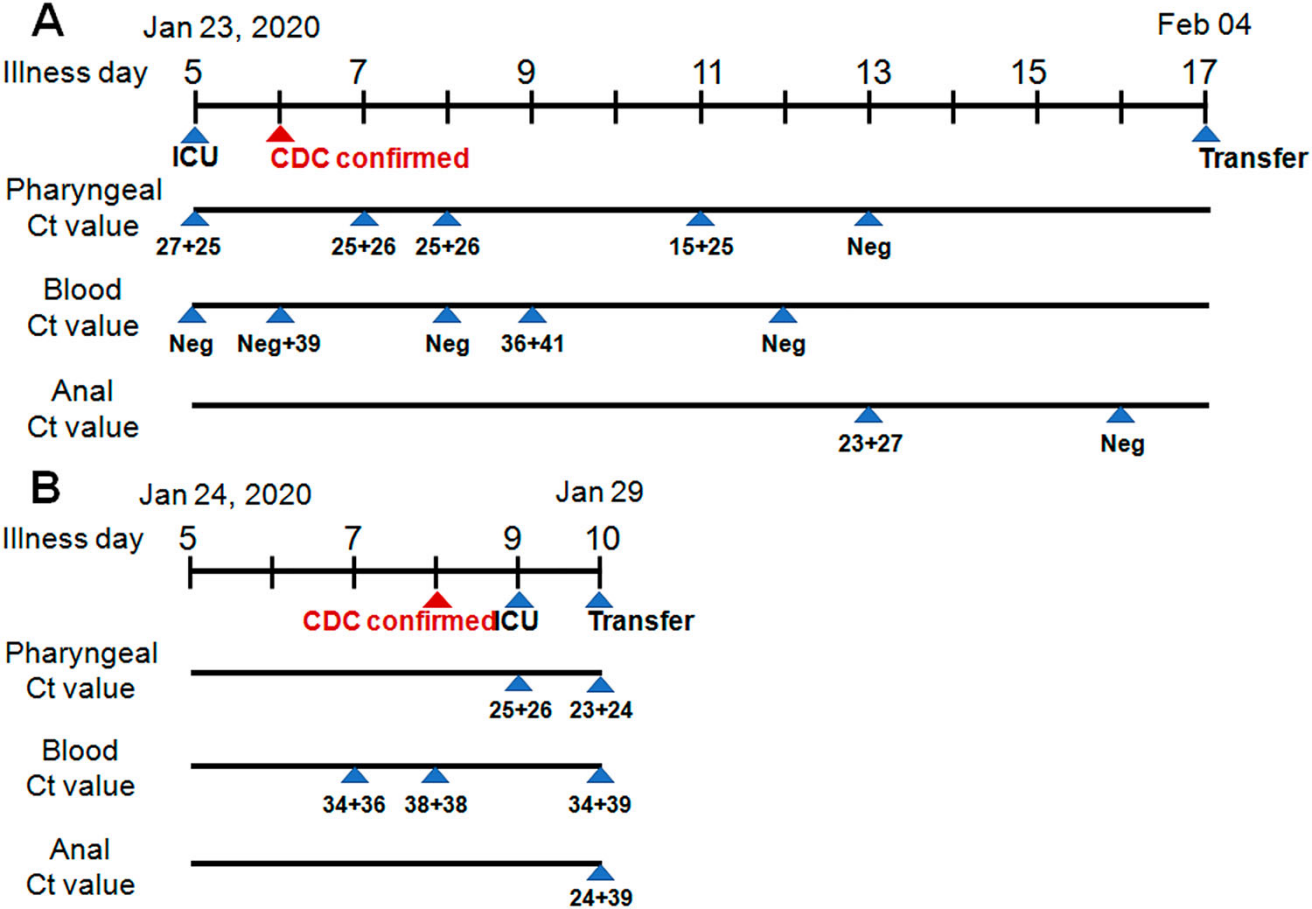
Diagram of viral detection in patient 1 (A) and 2 (B) with both detectable virus RNA in blood and anal swab. Illness day represents the patients with clear clinical symptoms such as fever, dry-coughing is labelled over the first line. ICU, CDC confirmed, and transfer indicates that the patient is in the ICU ward, CDC confirmation of viral positive, and transfer of patient out from ICU ward to another hospital to receive a higher level of medical care, respectively. Ct value from the pharyngeal, blood and the anal swab is shown.

Patient 2 (Figure 2(B)), who had a clear infection history and started fever 5-day ago and dry coughing 2-day ago, was admitted with clinically highly suspect of 2019-nCoV infection, considering the radiographical diagnosis which indicated clear pneumonia in the bilateral lung lobes. The virus was detected in his blood on illness day 7 (Ct = 34 + 36) and 8 (Ct = 38 + 38). His infection was also informed by the CDC on day 8. Because his disease advanced very fast, he was transferred to the ICU ward for special medical care requirements on day 9, on which day high titers of virus (Ct = 25 + 36) were detected in the pharyngeal sample. Importantly, virus RNA was detected in all pharyngeal (Ct = 23 + 24), blood (Ct = 34 + 39) and anal (Ct = 24 + 29) samples on day 10. He was transferred out to another hospital after an emergency expert consultation.

Finally, we described here the four patients with detectable serum viral RNA. Patient 3 (Figure 3(A)) was transferred to the ICU directly on illness day 11 because of his severe condition, the 2019-nCoV virus was laboratory detected both in pharyngeal (Ct = 30 + 30) and blood samples (Ct = 37 + 39) on day 12, And his infection was confirmed by CDC on day 13. Pharyngeal samples were PCR positive on days 14 and 17 and became negative on day 22. Patient 4 (Figure 3(B)) was transferred to the ICU ward on the illness day 6 with a CDC confirmation. His disease advanced pretty fast and became severe on day 7 and he was transferred to ICU after his blood sample was detected to be virus-positive (Ct = 32 + 37). On day 9, he was transferred out. Patient 5 (Figure 3(C)) was admitted on illness day 4 and his blood sample was virus-positive (Ct = 38 + Neg) on day 6. Her disease progressed rapidly to a severe stage within the next 3 days. Patient 6 (Figure 3(D)) with a clear history of virus infection was confirmed to be infected on infection day 7. Viral RNA was detected in his blood sample on day 9, one day ahead of his transfer into ICU. As his condition worsens, he was transferred out on day 13.

**Figure 3.**
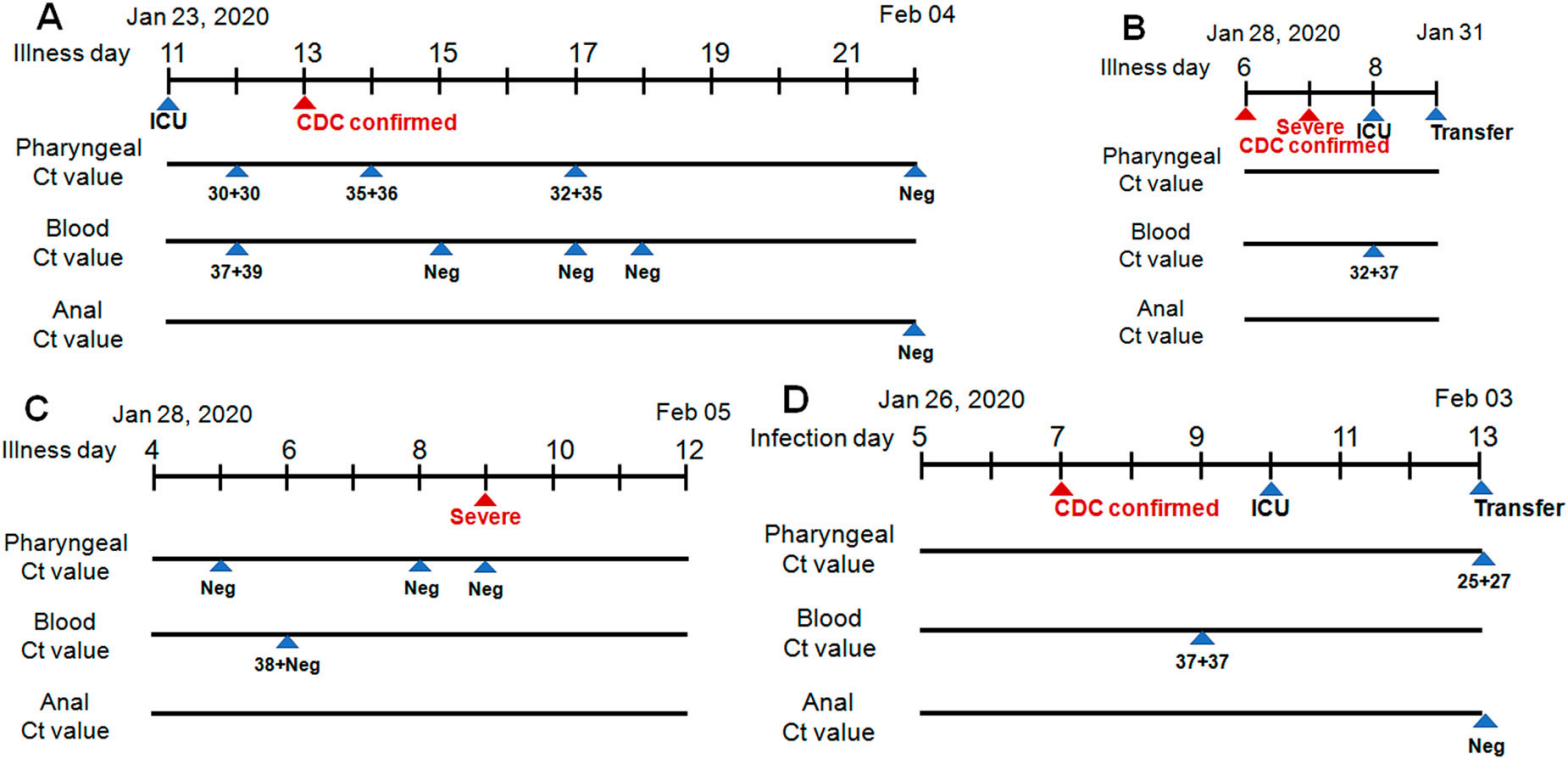
Diagram of viral detection in Patient 3 (A), 4 (B), 5 (C) and 6 (D) with both detectable virus RNA in blood. Labels are identical to those in Figure 2. Severe represents that the patient is diagnosed to be severe symptoms (B). Infection day represents the patient with a clear time of infection (D).

In this retrospective study, we analyzed the PCR data of virus detection in different tissues in our laboratory. Firstly, our observation indicated that the presence of viral RNA outside of the respiratory tract might herald the severity of the disease and alarm the requirement of special care. In the blood test cohort, all the 6 infected patients were in (or later progressed to) severe disease stage when serum viral RNA became detectable, which showed a significant difference compared to the blood negative group (*p* = 0.0001). Patient 2 (Figure 2(B)), 5 (Figure 3(C)) and 6 (Figure 3(D)) all had detectable viral RNA in the serum before they progressed to the clinical severe symptom stage. Unfortunately, we missed the earlier time points of patient 1 (Figure 2(A)) and 3 (Figure 3(A)) who were directly admitted to ICU on transfer to our hospital because of severe condition, of patient 4 (Figure 3(B)) who had serum sample collected one day post the diagnosis of severe illness. We, fortunately, observed high serum viral load in serum within their severe illness stage. In the anal swab cohort, we found that the presence of virus RNA in the anal digestive tract was also positively correlated with disease severity (*p* = 0.0102). The 3 patients detected with anal virus RNA but in mild stage should be monitored whether they will progress to the severe stage. We have summarized the information of approximately 70 percent of the patients in Guangzhou city, and the study represented nearly the whole picture of this region. However, the virus outbroke in such an emergence, allowing no delay in waiting for more patients to further confirm the findings.

Secondly, a high concentration of viral RNA in anal swabs suggested the digestive tract might be one extra-pulmonary site for virus replication. For patient 1, a high concentration of viral RNA (Ct = 23 + 27, on day 13) was detected in anal swab but not in pharyngeal (the same day) and blood (1 d ahead). For patient 2, higher concentrations of viral RNAs were detected in anal swab (Ct = 24 + 39) and pharyngeal swab (Ct = 23 + 24) than in the blood (Ct = 34 + 39) on the same day. Angiotensin-converting enzyme 2 (ACE2) still is one of the receptors for 2019-nCoV attachment and entry [2]. Intensive structural analysis of the S protein of 2019-nCoV with the SARS-Coronavirus suggested that several critical residues in the viral spike protein might confer favourable interaction with human ACE2 [7]. Of note, ACE2 is also abundantly present in humans in the epithelia of the small intestine besides the respiratory tract and is ubiquitously present in endothelial cells [8], which might provide possible routes of transmission, and might account for the high transmission capacity of the new virus. We propose that rampant coronavirus replication in pulmonary alveolus results in the breakdown of the alveolar vessel and the subsequent virus leakage into the blood flow, through which the virus is disseminated across the whole body. Then the virus succeeds in establishing reinfection in the digestive tract by using the highly expressed ACE2 receptor, which exacerbated the disease vice versa. Bat originated coronavirus was found to replicate in the swine digestive tract recently, also suggesting the potential replication possibility in the human digestive tract [9]. Nevertheless, confirmation of virus transmission through the digestive tract warrants further virus isolation from the anal swab in high safety level lab.

Unfortunately, in our study, we did not collect stool samples from patients and did not pursue viral RNA in the stool. But we believe the existence of virus RNA in the stool samples from these patients because that a large amount of viral RNA was detected in anal swabs and that viral RNA had also been detected in a case reported from the United States [10]. Also, we didn’t collect sputum and bronchoalveolar lavage fluid for virus detection because that the dry coughing characteristic of patients infected with 2019-nCoV prevents producing enough amount of sputum and that bronchoalveolar lavage fluid collection requires a sophisticated operation which increases virus exposure possibility of care providers to high concentrations of virus-containing aerosol.

In summary, we find that the presence of viral RNA in the blood and anal swab is positively correlated with the severe disease stage and that early monitoring of virus RNA in blood and the digestive tract on top of the respiratory tract might benefit the disease prediction.
